# Deciphering the Immunological Phenomenon of Adaptive Natural Killer (NK) Cells and Cytomegalovirus (CMV)

**DOI:** 10.3390/ijms21228864

**Published:** 2020-11-23

**Authors:** Samantha Barnes, Ophelia Schilizzi, Katherine M. Audsley, Hannah V. Newnes, Bree Foley

**Affiliations:** 1 Telethon Kids Institute, University of Western Australia, Perth Children’s Hospital, Nedlands, WA 6009, Australia; samantha.barnes@telethonkids.org.au (S.B.); ophelia.schilizzi@telethonkids.org.au (O.S.); katherine.audsley@telethonkids.org.au (K.M.A.); hannah.newnes@telethonkids.org.au (H.V.N.); 2 School of Biomedical Sciences, The University of Western Australia, Crawley, WA 6009, Australia

**Keywords:** natural killer cells, cytomegalovirus, viral infection, transplantation, vaccination, cancer immunotherapy

## Abstract

Natural killer (NK) cells play a significant and vital role in the first line of defense against infection through their ability to target cells without prior sensitization. They also contribute significantly to the activation and recruitment of both innate and adaptive immune cells through the production of a range of cytokines and chemokines. In the context of cytomegalovirus (CMV) infection, NK cells and CMV have co-evolved side by side to employ several mechanisms to evade one another. However, during this co-evolution the discovery of a subset of long-lived NK cells with enhanced effector potential, increased antibody-dependent responses and the potential to mediate immune memory has revolutionized the field of NK cell biology. The ability of a virus to imprint on the NK cell receptor repertoire resulting in the expansion of diverse, highly functional NK cells to this day remains a significant immunological phenomenon that only occurs in the context of CMV. Here we review our current understanding of the development of these NK cells, commonly referred to as adaptive NK cells and their current role in transplantation, infection, vaccination and cancer immunotherapy to decipher the complex role of CMV in dictating NK cell functional fate.

## 1. Introduction

Cytomegalovirus (CMV) has an interesting and diverse relationship with the human immune system, co-evolving side by side for millions of years to produce a finely tuned symbiotic relationship under normal homeostatic conditions. However, while immunocompetent individuals rarely present with symptoms, CMV infection remains a serious threat to immunocompromised individuals such as transplant recipients and is the most common congenital infection that can lead to significant neurological deficiencies in newborns [1]. Natural killer (NK) cells play an important role in combating CMV infection, which has resulted in a dynamic interplay between NK cells and CMV evasion mechanisms. Arguably one of the most important consequences of this relationship is the emergence of a subset of NK cells known as adaptive NK cells. To date only identified in the context of CMV infection, the discovery of these NK cells has played a significant role in advancing our understanding of NK cell function and their ability to bridge the divide between innate and adaptive immune responses. Furthermore, adaptive NK cells have emerged as important players across several contexts from viral infections and vaccination to transplantation and cancer immunotherapy.

## 2. Biology of NK Cells

Discovered in the mid 1970s, NK cells are categorized as CD56^+^ CD3^−^ cells that are unique in their ability to kill target cells without prior antigen sensitization [2]. This feature is critical for the rapid elimination or containment of infection, allowing the recruitment and activation of the adaptive immune system for a specific attack and the development of immune memory. NK cells are commonly split into two major subtypes based on the density of CD56. These subtypes are defined broadly by their distinct functions, delineated generally by cytotoxic effector capacity (CD56^dim^) and immunoregulatory cytokine production (CD56^bright^) [3]. CD56^bright^ NK cells produce cytokines such as interferon gamma (IFNγ), tumor necrosis factor alpha (TNFα) and granulocyte-macrophage colony-stimulating factor (GM-CSF), soluble factors that are necessary for the recruitment of other immune cells during the initial innate immune response [4]. Whilst CD56^dim^ NK cells are similarly capable of secreting cytokines, they are distinguished by their ability to induce target cell apoptosis through the release of lytic granules containing perforin and granzymes [5]. As such, NK cells play an important role in bridging the innate and adaptive immune systems, regulating the immune response to virally infected and tumorigenic cells.

The capacity of NK cells to recognize infected cells is determined by a balance of germline-encoded activating and inhibitory receptors. The combination of signals received by these receptors determines whether an NK cell is activated by the target cell. Inhibitory receptors on NK cells play an important role in self-recognition and NK cell education [6]. Prominent inhibitory receptors on NK cells are CD94/NKG2A, which recognizes the non-classical human leukocyte antigen (HLA)-E molecule, the killer immunoglobin-like receptors (KIRs) that recognize allelic epitopes present in certain HLA-A, -B and -C alleles and the leukocyte immunoglobulin-like receptors (LIRs) such as LIR-1 (CD85j) which binds HLA class I alleles with varying affinities [7]. NK cells gain functional competency during development through a process known as NK cell education [8]. When an educated NK cell encounters its HLA ligand on a target cell its activity is inhibited. Comparatively, the absence of this inhibitory signal will trigger activation through dominating activating signals, termed ‘missing self’ [6]. Activating signals are received through a host of receptors, such as NKG2D, NKG2C, activating KIRs and the natural cytotoxicity receptors (NCRs); NKp30, NKp44 and NKp46, which bind to ligands upregulated on stressed or virally infected cells such as major histocompatibility complex class I chain-related A (MICA), MICB, UL16 binding proteins (ULPBs), B7-H6 and haemagglutinin [9,10]. The activating receptor FcγRIIIA (CD16) is able to induce antibody-dependent cellular cytotoxicity (ADCC), an apoptotic pathway where target cells are opsonized with IgG, triggering the release of perforin and granzyme B [11]. Activating receptors are often associated with co-receptors necessary for triggering this cytotoxic activity within the NK cell such as DNAX accessory molecule-1 (DNAM-1), CD96, NK-T-B antigen (NTBA) and CS1 (CD319). Together with inhibitory signals, NK cells rely on activating signals expressed by target cells to determine the degree of response to their cellular environment. This degree of response may differ between individuals as a function of the polymorphic nature of the varied NK cell receptor repertoires [12], in addition to lifetime exposure to pathogenic and other environmental factors [13].

## 3. NK Cells and CMV

A well-established case study where dynamic NK cell receptors and infection interact is in the context of CMV. CMV is a member of the Herpesviridae family of viruses and affects between 50–98% of human populations, depending on geographic locations and socio-economic backgrounds [14]. Once infected, the virus remains latent in the host for life with frequent reactivations. Although generally benign, CMV is damaging for immunocompromised individuals and newborns, with individuals deficient in either the function or number of NK cells experiencing severe disease, sometimes resulting in death [15]. In healthy individuals, CMV avoids elimination by the immune system through a variety of escape mechanisms likely developed over millions of years of coevolution with humans [16].

CMV encodes a set of genes that mediate these escape mechanisms, mostly through targeting HLA molecules on the surface of the infected cell by either interfering with the expression of HLA or encoding for HLA homologues [17]. US2, US3, US6 and US11 are inhibitory unique short region proteins expressed at different stages of infection [18]. US2 and US11 trigger proteasomal degradation of the HLA molecule, US3 promotes retention of the protein in the ER, while US6 inhibits peptide loading by blocking the transporter protein, TAP, binding to ATP [19]. Together, these proteins ensure the downregulation of HLA molecules to avoid CD8^+^ T cell detection. Under normal conditions, this downregulation of HLA molecules would trigger the ‘missing self’ pathway of NK cell recognition and leave CMV-infected cells vulnerable to detection and elimination. However, CMV has evolved to express certain HLA homologues and peptide homologues to inhibit NK cell activation. The first CMV-encoded immune-evasive protein reported was UL18, a homologue of HLA [20]. Expressed in its place, UL18 has a high binding affinity to the NK cell inhibitory receptor LIR-1 in humans, up to 1000-fold more than when bound to its endogenous ligand, HLA class I [21]. 

Additional inhibitory mechanisms also exist, including the production of proteins UL16 and UL40. UL16 acts by inhibiting the production of MICA, MICB and ULBPs, ligands for the activating receptor NKG2D [22]. UL40 is a peptide produced by CMV homologous to the HLA-E binding protein [23]. UL40 allows for expression of HLA-E on the surface of infected cells by promoting its loading in a TAP-independent manner, bypassing inhibition by US6 [24]. UL40 bound to HLA-E binds to the inhibitory receptor NKG2A, providing the CMV infected cell with an effective NK cell escape mechanism [25]. However, the activating receptor NKG2C also recognizes HLA-E, the ramifications of which will be discussed in detail below. This interplay between the host and CMV allows CMV to persist in the body and shape the ensuing immune response for life. 

## 4. History of Adaptive NK Cells

CMV plays a unique role in shaping the NK cell repertoire and driving the expansion of subset(s) of NK cells with memory-like properties, now routinely known as adaptive NK cells. First described in the context of mouse CMV (MCMV), these NK cells can expand and contract following infection [26]. NK cells expressing the activating receptor Ly49H directly recognize the MCMV protein, m157. Following MCMV infection, these NK cells proliferate over 100-fold in the spleen and 1000-fold in the liver before contracting once disease is cleared. Viral specific Ly49H^+^ NK cells however do not return to their pre-infection baseline and instead are maintained at an elevated level for several months. Upon rechallenge Ly49H^+^ NK cells respond and degranulate far more rapidly than CMV naïve NK cells and produce significantly more cytokines to protect against disease progression. 

In humans, adaptive NK cells are most commonly associated with expression of NKG2C. Guma and colleagues [27] first reported the observation that NK cells expressing NKG2C were increased in CMV seropositive (CMV^+^) healthy donors. A similar association was not detected in patients who were herpes simplex virus (HSV) or Epstein-Barr virus (EBV) seropositive, suggesting the expansion of NKG2C was specific to CMV infection and not to other Herpesviridae infections. In addition, NK cells expressing NKG2C had lower expression of the two activating receptors, NKp30 and NKp46 and increased proportions of KIR and LIR-1 expression. Building on these observations, Guma and colleagues [28] later demonstrated that NK cells expressing NKG2C preferentially expand following co-culture with CMV-infected fibroblasts using the AD169 or Towne strains of CMV. This expansion did not occur with virus alone nor was there any expansion of NKG2C^+^ NK cells in CMV seronegative donors. Importantly, blocking NKG2C on HCMV donors decreased NK cell expansion demonstrating that NKG2C was directly involved in mediating this phenomenon, although the mechanisms driving this remained unclear. 

Unlike in MCMV, where there is a distinct population of MCMV-specific Ly49H NK cells that expand, contract and respond preferentially upon rechallenge [26], how CMV shapes the NK cell receptor repertoire in humans is more complex. Nevertheless, multiple studies have provided evidence for the possibility of a memory-like NK cell population in humans in the context of CMV infection [29,30,31,32,33,34,35]. NK cells co-expressing NKG2C and CD57 were first associated with CMV infection in solid organ transplant (SOT) recipients [33]. CD57 is expressed on mature NK cells and typically associated with high effector function [36]. Following CMV infection in these SOT recipients, CD57^+^NKG2C^+^ NK cells preferentially expanded over time. In the context of hematopoietic stem cell transplantation (HSCT), we similarly observed an expansion of NK cells expressing NKG2C with increased acquisition of CD57 over time [32]. During acute CMV reactivation in recipients of umbilical cord blood (UCB) transplants, NKG2C^+^ NK cells preferentially expand, peaking at four weeks post-infection. This population remained a significant proportion of each patient’s NK cell receptor repertoire at one year post-transplant, accounting for approximately 50% of their NK cells. In addition, these NKG2C^+^ NK cells produced significantly more IFNγ compared to NKG2C^−^ NK cells. NK cells are the first lymphocyte to reconstitute following UCB HSCT; however, they present with an immature phenotype and overall poor effector function that can take up to a year to be restored [37]. CMV reactivation appeared to dramatically accelerate the maturation of these NK cells, as demonstrated by a rapid decline in NKG2A expression, acquisition of KIR, CD57 and enhanced effector function [32]. Furthermore, increased mRNA expression of T-bet and IFNγ was detected six months and one year post-transplant, indicating their potential to respond more rapidly with cytokines and acquire a memory-like phenotype. In a follow-up study, we identified that NKG2C^+^ NK cells expand even in the absence of clinically-detectable CMV viraemia in CMV but only if both the donor and recipient were CMV^+^ [31]. This did not occur if the recipient was CMV seronegative, suggesting that the continued persistence of NKG2C^+^ NK cells requires the presence of latent (subclinical) expression of CMV antigen. Furthermore, in recipients who received a transplant from a CMV^+^ donor, we demonstrated the potential of NKG2C^+^ NK cells to represent memory-like NK cells with heightened effector function and expansion following CMV reactivation. Collectively, these studies demonstrate the profound effect CMV has on the NK cell receptor repertoire and the potential for memory-like NK cells to exist in humans. 

The ability of CMV to imprint on the NK cell receptor repertoire was highlighted in a large cohort study of over 200 healthy donors [29]. In elegant detail, Beziat and colleagues demonstrated the ability of CMV infection to skew NK cell receptor repertoires towards an abundance of NKG2C^+^ CD57^+^ KIR^+^ self-educated NK cells. This general phenotype is strongly associated with those NK cells exhibiting the highest functional potential against target cells. In addition, other NK cell subsets identified lacked NKG2C and expressed activating KIR, suggesting NK cells expressing activating KIR also expand in the context of CMV infection. Indeed, adaptive NK cells have been shown to expand in individuals who lack NKG2C, clearly demonstrating that NKG2C is not required for the expansion of adaptive NK cells [38,39,40,41]. Beyond surface phenotypes, we and others have reported on the downregulation of the adaptor molecule FcεRIγ, the kinase Syk, the intracellular adaptor EAT-2 and the transcription factor PLZF (promyelocytic leukemia zinc finger) in CMV^+^ individuals [35,42]. Similar to our previous observations in HSCT recipients, NK cells lacking FcεRIγ, EAT-2 and Syk expanded during the first year after transplantation. However, what was most striking about these studies was the link between CMV infection and epigenetic modification of the NK cells, a notion first identified in 2014 with the identification of CMV infection epigenetically imprinting on the IFNG locus [34]. Compared with canonical NK cells, memory-like NK cells possess a methylation profile far more similar to CD8^+^ T cells [35]. This, coupled with their memory-like phenotype, led to their classification as adaptive NK cells, which to date have only been identified in CMV^+^ individuals. In the following sections we will provide an update on the development of adaptive NK cells, followed by their role in transplantation, viral infections, vaccination and cancer immunotherapy.

## 5. Current Status on the Development of Adaptive NK Cells

For many years there were two main overarching questions surrounding the development of adaptive NK cells: how is CMV driving the expansion of adaptive NK cells and why are these cells only found in approximately one-third of CMV^+^ donors? Following the first reports of an association between NKG2C and CMV, Guma and colleagues [28] attempted to determine how NKG2C^+^ NK cells were recognizing infected target cells. NKG2C recognizes the non-classical class I allele, HLA-E [43]. In CMV infection, HLA-E is not downregulated like other class I alleles and can be loaded with a peptide derived from the leader sequence of the CMV UL40 protein [23]. Presumably this has evolved to inhibit NK cell function through binding of HLA-E to the inhibitory NKG2A receptor. However, when using UL40 mutated viral strains there was only a minor effect on the expansion of NKG2C^+^ NK cells. It was not until 2018, when Hammer and colleagues [44] elegantly demonstrated the ability of the UL40 CMV peptide loaded in HLA-E to drive the expansion of NKG2C^+^ adaptive NK cells, that the role of CMV peptides was finally elucidated. Importantly, not all UL40 CMV peptides were equal in their ability to drive expansion. The VMAPRTLFL (found in ~1% of clinical isolates) peptide had the greatest ability to activate NKG2C^+^ adaptive NK cells whereas the VMAPRTLIL (found in ~40% of clinical isolates) and VMAPRTLVL (found in ~12% of clinical isolates) required co-stimulation through CD2-LFA-3 to achieve similar levels of activation. Arguably the most striking finding in this seminal publication was the ability to generate adaptive NK cells from CMV seronegative donors, which had previously never been reported. Peptide alone was not sufficient to drive expansion of NKG2C^+^ NK cells from CMV seronegative donors but required CD2 co-stimulation and was even greater in the presence of the proinflammatory cytokines interleukin (IL)-12 and IL-18. In addition to an expanded NKG2C^+^ population, these NK cells also exhibited other features of adaptive NK cells such as downregulation of FcεRIγ and hypomethylation of the IFNG locus. Hammer and colleagues [44] extended their findings to a cohort of HSCT recipients with CMV reactivation. The peptide-encoding UL40 region of the infecting viral strain was assessed for each patient and compared with the presence and/or expansion of adaptive NK cells. Recipients infected with strains harboring the VMAPRTLFL peptide expanded NK cells with an adaptive phenotype, whereas of those patients infected with strains harboring the VMAPRTLIL peptide, the presence of adaptive NK cells was far more varied. Interestingly, the VMAPRTLFL peptide can also be derived from the leader sequence of HLA-G, with Rolle and colleagues suggesting a role for increased HLA-G expression during CMV infection as another driver of the expansion of adaptive NK cells [45]. Clearly CMV peptide plays a significant role in the expansion of adaptive NK cells, however due to the great variation in the responses seen, other factors are likely to also be involved. Furthermore, adaptive NK cells do not all express NKG2C, which strongly suggests that other viral or potentially self-peptides also contribute to the generation of adaptive NK cells. Studies to identify these peptides, for example ones that may be recognized by activating KIR, are certainly warranted and will provide another important piece in the puzzle to understanding the unique relationship between CMV and adaptive NK cells. 

**Figure 1:**
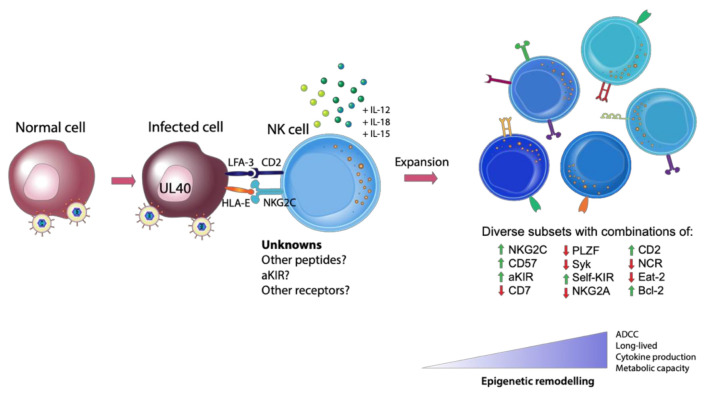
Generation of Adaptive natural killer (NK) cells. The expansion of adaptive NK cells requires three signals: (1) appropriate receptor engagement in the context of a viral peptide (such as HLA-E and NKG2C, with other unknown viral or self-peptides and receptors likely involved), (2) co-stimulation and (3) proinflammatory cytokines. This engagement with a virally infected cell leads to the generation of diverse, highly functional subsets of NK cells with differing degrees of epigenetic remodeling.

Additional research is also required to elucidate how CMV infection remodels the epigenetic landscape of adaptive NK cells. We know that at least three signals are required to induce epigenetic changes that result in the formation of adaptive NK cells: ligand engagement in the context of an appropriate peptide, co-stimulation and proinflammatory cytokine stimulation [44] (Figure 1). The peptide is crucial as proinflammatory cytokines alone do not generate adaptive NK cells, rather they generate a population of cytokine induced memory-like NK cells with their own distinct phenotype and functional capacity [46]. It would be interesting to examine whether every NK cell has the potential to become an adaptive NK cell or whether this is exclusive to a specific subset. One may speculate the existence of a population of NK cells that are more poised to undergo epigenetic remodeling than other NK cells, awaiting a strong engagement with a viral peptide during infection to drive them towards an adaptive phenotype. Answering these questions will be challenging, yet a deeper understanding of the potential of NK cells to gain an adaptive phenotype will allow researchers greater capacity to exploit these cells for future NK cell immunotherapies. 

## 6. Current Status on the Role of Adaptive NK Cells in Transplantation

Expansion of adaptive NK cells was first reported in transplant recipients due to the high prevalence of CMV infection following transplantation and the ability to track developing NK cells over time [30,31,32,33]. Overall, CMV reactivation following transplantation remains a serious and potentially life-threatening complication [47]. However, several studies have reported positive correlations between the incidence of CMV reactivation and reduced leukemic relapse. Elmaagacli and colleagues [48] reported that early CMV reactivation (prior to day 100) was associated with reduced risk of leukemic relapse in 266 acute myeloid leukemia (AML) patients. The risk of relapse 10 years post-transplant was 9% in patients with reactivated CMV compared to 42% in those without. Similar studies have since reported further associations with CMV reactivation and risk of relapse following HSCT [49,50,51,52]. Conversely, several studies have reported negative or no associations between CMV reactivation and relapse [53,54,55]. In particular, a large study from the Centre for International Blood and Marrow Transplant Research (CIBMTR) retrospectively analyzed the association of CMV reactivation with transplant outcomes in 9469 patients found an increased risk of transplant-related mortality following CMV infection [56]. There are many potential factors that may influence these discordant responses such as transplant protocols, detection of CMV infection and anti-viral treatment protocols that are beyond the scope of this review article. However in the context of adaptive NK cells, expansion of adaptive NK cells has been associated with better clinical outcomes in HSCT recipients [57,58]. What role adaptive NK cells are playing in mediating protective outcomes in HSCT recipients remains unknown. NK cells are well-known for their ability to mediate the graft versus leukemia effect [59]. Whether adaptive NK cells have the capacity to directly target residual leukemic cells or support the elimination of these leukemic cells needs to be formally evaluated. Furthermore, the role adaptive NK cells play during CMV infection is also unclear. In a small cohort of 58 patients, expansion of adaptive NK cells was correlated with CMV reactivation but not CMV disease (as defined by the severity of the disease resulting in pneumonitis or gastrointestinal disease) [60]. In fact, patients with severe CMV disease had lower overall NK cell function one month following CMV viraemia. This suggests that the severity of CMV disease influences the expansion of adaptive NK cells but also raises the possibility that adaptive NK cells may play a role in controlling the severity of CMV disease. Similarly, in the context of SOT, adaptive NK cells have been associated with reduced incidence of CMV viraemia in kidney transplant recipients [61]. Conversely, increased proportions of adaptive NK cells in bronchial lavage fluid has been associated with chronic lung allograft dysfunction and even death, suggesting that adaptive NK cells responding to CMV infection may cause considerable life-threatening collateral damage in transplant recipients. This dichotomous interplay between CMV and adaptive NK cells following transplantation suggests a critical balance between both the virus and the NK cell response to the virus. Why adaptive NK cells mediate this dichotomous response is unclear. Multiple factors such as the viral strain, severity of disease, degree of expansion of adaptive NK cells and other host factors all play a role in determining transplant outcomes. A deeper understanding of these factors and how they interact are certainly warranted to provide new approaches to improve outcomes for transplant recipients.

## 7. Current Status on the Role of Adaptive NK Cells in Viral Infection

Healthy individuals are typically able to control CMV infection through an effective multifaceted immune response. However, it remains unknown whether this adaptive NKG2C^+^ NK cell population is a significant effector of anti-CMV immunity, as to date the majority of evidence supporting this role is circumstantial. In one case study, expansion of NKG2C^+^ NK cells was observed in an infant with a T-B-NK^+^ severe combined immunodeficiency disorder phenotype following the spontaneous resolution of HCMV-induced gastroenteritis [62]. However, NKG2C^+^ NK cell expansions are typically observed in only 1/3 of individuals following CMV infection [35] and are heavily reliant upon recognition of strain-specific UL40 peptides and pro-inflammatory signals [44]. Individuals with a deletion in the NKG2C gene (KLRC2) present a unique opportunity to study the role of NKG2C^+^ NK cells in CMV infection. Deletion of the 16 Kb region encompassing the KLRC2 gene is relatively common, with a predicted 34% heterozygous and 3–5% homozygous deletion frequency in Japanese and European populations [63,64]. Although NKG2C deletion has been reported as a risk factor for CMV infection [65], analysis of congenital CMV infection revealed no significant difference in the frequency of heterozygous NKG2C deletion between CMV-infected children and uninfected controls [66]. Additionally, the homozygous NKG2C deletion was found in one uninfected control individual. Further investigation into the adaptive NK cell response to CMV infection is therefore warranted. 

Like CMV, chronic human immunodeficiency virus (HIV) infection has also been reported to alter the composition and effector function of the NK cell compartment. The observed downregulation of activation receptors, upregulation of inhibitory receptors and emergence of an abnormal CD56-CD16^+^ NK cell subset are thought to collectively contribute towards the dysfunctional and exhausted NK cell phenotype associated with the inflammatory environment of chronic HIV [67]. Though most of the work to date has focused on adults, conflicting observations have recently been reported in pediatric patients undergoing treatment for HIV [68]. Interestingly, Mahapatra and colleagues reported elevated expression levels of NK cell activation receptors such as NKp40 and NKG2C and other stimulation receptors such as CD2 and CD11c in HIV-infected children compared to uninfected controls. However, as this was the first study to investigate the impact of HIV infection on the NK cell repertoire in pediatric patients, further work is needed to elucidate the mechanisms driving these changes. Given the high prevalence of CMV seropositivity amongst HIV^+^ individuals (>90%), many of the changes observed in the NK cell repertoire following HIV infection are likely confounded by co-infection with CMV [69]. Guma and colleagues [70] were the first to implicate underlying CMV infection with the expansion of NKG2C^+^ NK cells in HIV^+^ patients. These expansions occur independently of HIV viral load [71], though the requirement for active CMV infection remains unclear. Interestingly, the presence of these CMV-driven adaptive NK cells may contribute to better patient outcomes for those with concomitant HIV infection. Strong anti-HIV activity has been observed in NKG2C^+^ NK cells from HIV^+^ but not HIV^-^ individuals, suggesting a role in anti-HIV immunity [72]. A higher proportion of NKG2C^+^ NK cells has also been associated with a lower HIV viral setpoint [72] and better responses to antiretroviral therapy during primary HIV infection [73]. Furthermore, CMV^+^ patients with homozygous deletion of the NKG2C gene are at a greater risk for rapid disease progression once HIV occurs [38,73,74]. 

Whilst clearly important in HIV infection, the impact of this CMV-driven NKG2C^+^ NK cell population remains unclear in the context of other infections. It has been hypothesized that CMV infection may prime this population of NK cells for efficient expansion following subsequent viral infection. Indeed, increased frequencies of NKG2C^+^ NK cells have been observed in individuals infected with hantavirus [75], chikungunya virus [76], dengue virus [77] and viral hepatitis B and C [78], though CMV seropositivity remains a pre-requisite. EBV co-infection was also initially thought to drive expansion of NKG2C^+^ NK cells in CMV^+^ individuals [79], however further analysis revealed this observation was actually a result of an EBV-driven reduction in the number of CD56^dim^CD16^+^ NK cells [80]. Adaptive NK cells are known to harbor an enhanced capacity for ADCC and as such have greater potential for a broad antiviral response against infected cells. NKG2C^+^ NK cells have also been shown to mediate ADCC against malaria-infected red blood cells [81]. CMV-driven adaptive NK cells were observed to provide a protective effect, with greater frequencies of NKG2C^+^ NK cells associated with prolonged time until malaria infection, reduced severity of symptoms and reduced parasitic load [81]. In contrast, the presence of NKG2C^+^ NK cells appear to have negative consequences for other co-infections. For example, the reduced signaling capacity and NKp46 expression in adaptive NK cells may inhibit co-stimulation between NKp46 and viral haemagglutinin, potentially compromising the NK cell response to influenza virus [82]. Emerging data from the ongoing COVID-19 pandemic have also associated CMV seropositivity with poorer patient outcomes. Increased frequency of NKG2C^+^ NK cells was linked to greater disease severity, with approximately 2/3 of CMV^+^ severe COVID-19 patients demonstrating adaptive NK cell expansion compared to 1/3 of CMV^+^ healthy controls and even fewer CMV^+^ moderate COVID-19 patients [83]. Interestingly, this increased proportion of NKG2C^+^ NK cells in COVID-19 patients was independent of CMV reactivation and did not correlate with serum levels of anti-CMV IgG. Understanding the mechanisms that drive adaptive NK cells to mediate these diverse and potentially negative outcomes is clearly required to harness their therapeutic potential.

## 8. Current Status on the Role of Adaptive NK Cells in Vaccination

Vaccination facilitates the control, elimination and eradication of infectious diseases. Although vaccines have traditionally focused on eliciting an adaptive immune response, emerging evidence suggests that NK cells are also important effectors of post-vaccination immunity. In the context of a vaccine recall response, NK cells demonstrate a heightened IFNγ response and comprise up to 70% of all IFNγ-producing cells in the first 12–24 h following vaccination [84]. NK cells may also contribute to both the induction and effector phases of post-vaccination immunity via two distinct mechanisms: activation by IL-2, derived from antigen-specific CD4^+^ T cells or by mediating ADCC through cross-linking of CD16 by antigen-antibody immune complexes [84,85,86]. However, sensitivity to these signals is influenced by several factors including the NK cell differentiation state and the CMV infection status of the individual [87]. As new diseases continue to emerge, a greater understanding of the factors that govern the NK cell response to vaccination will be crucial for the development of new and more effective vaccines.

The impact of CMV infection on the NK cell vaccination response is complex. Increased frequencies of NKG2C^+^ NK cells have been observed in individuals who respond to influenza vaccination compared to non-responders [88]. Due to their enhanced capacity for ADCC, adaptive NK cells also positively contribute to the vaccination response against Ebola virus, contributing up to 25% of the post-vaccination NK cell-derived IFNγ response [89]. In contrast, several studies have also reported that CMV^+^ individuals exhibit an impaired in NK cell-derived IFNγ response to previously encountered vaccine antigens including influenza, whole cell pertussis and DTPiP [85,90,91]. CMV infection may also harbor broad consequences for vaccines that rely on IL-2 or other accessory cytokines to drive a vaccination response [92]. A reduction in the NK cell-derived IFNγ response to exogenous cytokines is well documented amongst CMV^+^ individuals [85,87,91]. In part, this is reflective of the CMV-driven shift towards the more mature CD56^dim^CD57^+^ NK cell phenotype and corresponding decrease in the frequency of the less-differentiated and intrinsically more cytokine-responsive CD56^bright^ and CD56^dim^CD57^−^ populations [85,91]. However, chronic CMV infection has been reported to reduce the cytokine responsiveness of all NK cell subsets and as such other mechanisms are also likely contributing to this effect [85,91]. Remarkably, several vaccines are able to boost the cytokine responsiveness of NK cells in CMV^+^ individuals. Influenza vaccination was shown to preferentially increase IL-12 and IL-18 stimulated NK cell responses in CMV^+^ but not CMV^−^ Europeans [91]. TIV and DTPiP vaccination achieved similar results in African populations, where CMV infection is near ubiquitous [90]. Increases in cytokine-driven NK cell IFNγ responses were also observed in European and African populations following BCG or yellow fever vaccination, respectively, though CMV serostatus was not investigated [93,94]. Although the extent to which adaptive NK cells participate in the vaccination response is unclear, it is plausible that high frequencies of these cells may impact the effectiveness of vaccination in CMV^+^ individuals. Strategies that take the adaptive NK cell subset into consideration may therefore prove beneficial in the development of new and improved vaccines. 

## 9. Current Status on the Role of Adaptive NK Cells in Cancer Immunotherapy

Due to their heightened effector function [29,31,32,35], enhanced capacity for ADCC [42,95,96], potential to be long-lived [35,95,97] and their clinical association with reduced leukemic relapse following HSCT [57,58], there is much interest in utilizing adaptive NK cells as an immunotherapy to treat cancer. For the treatment of leukemia, Liu and colleagues [98] demonstrated the potential of ex vivo expanded adaptive NK cells to eliminate pediatric acute lymphoblastic leukemia (ALL). NKG2C^+^ adaptive NK cells were expanded using feeder cells expressing HLA-E, however recognition of ALL cells in this study was not dependent on NKG2C recognition of HLA-E. Rather activating receptors such as DNAM-1 or NKG2D are likely involved [99]. This suggests that while NKG2C marks adaptive NK cells, its contribution to the recognition and elimination of cancer cells remains unclear. In support of this we have recently demonstrated that while NKG2C^+^ adaptive NK cells were associated with increased effector function against B cell ALL, NKG2C recognition of HLA-E was not involved in this increased function [100]. There is also rationale to exploit the enhanced ADCC potential of adaptive NK cells to generate improved responses against cancer when using monoclonal, bispecific and trispecific antibodies [96]. However, extrapolating adaptive NK cells into an adoptive cellular therapy is not without its challenges. Due to their more differentiated phenotype, adaptive NK cells proliferate poorly when using standard expansion protocols. Feeder cells expressing HLA-E have been used to enrich for NKG2C^+^ adaptive NK cells [98] yet it is unclear if the NKG2C^+^ NK cells are significantly expanding or rather it is the lack of NKG2A^+^ NK cells expanding due to inhibition through HLA-E. Another method to enhance expansion of adaptive NK cells involves the addition of the GSK3 inhibitor CHIR99021 to a feeder-free, IL-15 culture protocol [101]. GSK3 is a constitutively active serine-threonine kinase originally identified for its role in glucose metabolism, however has since been recognized in cell differentiation, survival and immune responses [102]. How GSK3 inhibition promotes the expansion of adaptive NK cells is unclear. Nevertheless, adaptive NK cells expanded in the presence of GSK3 inhibition significantly improved survival in a preclinical model of ovarian cancer [101]. Clinical trials are currently underway using this protocol to treat ovarian cancer (NCT03212964), AML (NCT03081780) and various solid cancers in combination with monoclonal antibody therapy (NCT03319459). Determining the optimal protocol to expand adaptive NK cells for adoptive cellular therapy is crucial to improve outcomes in patients with cancer. It is also worth speculating whether the serostatus of the recipient will influence the long-term potency and expansion of adaptive NK cells following adoptive transfer. Our identification of decreased adaptive NK cell expansion in CMV seronegative recipients suggests subclinical CMV antigen supports the persistence of adaptive NK cells [31]. If the recipient of adoptive cellular therapy using adaptive NK cells is CMV seronegative, how will this affect the potency and expansion of the transferred cells? Or will the transferred adaptive NK cells be able to quickly eliminate the cancer cells and are therefore not required to persist? These outcomes will not be known until the results from various clinical trials exploiting adaptive NK cells to treat cancer are published, which we await with eager anticipation. 

## 10. Concluding Remarks

**Figure 2:**
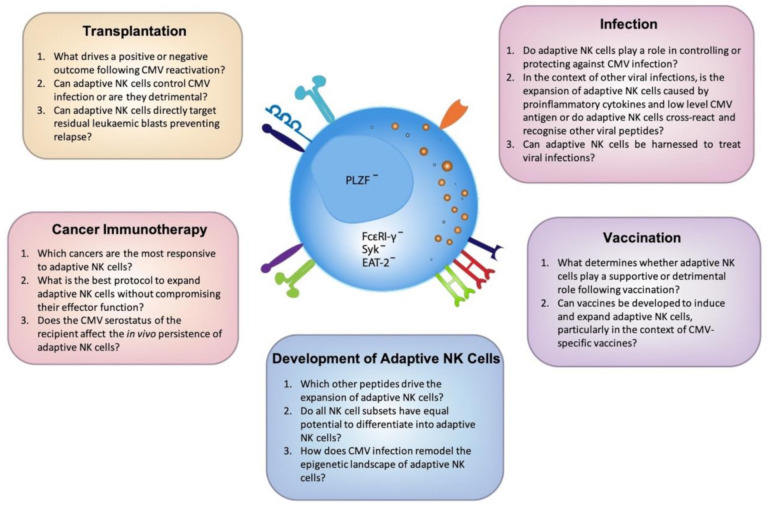
Outstanding questions to unlock the full potential of adaptive NK cells. Cytomegalovirus (CMV) has the unique ability to imprint on the NK cell repertoire resulting in the expansion of diverse, highly functional adaptive NK cells. A greater understanding of the mechanisms that drive the development of these cells is clearly warranted. Here we have identified the most pressing questions across a number of emerging fields which, upon further investigation, will elucidate the maximal clinical potential of adaptive NK cells.

The profound effect CMV has on modulating the human immune system remains a fascinating and mysterious immunological phenomenon. Why this commonly asymptomatic virus drives the expansion of NK cells that undergo significant epigenetic reprogramming is still not clear and understanding these drivers will only further increase the potential of these cells to be used therapeutically. Due to their increased functional capacity and adaptive-like features, including their potential to be long-lived, adaptive NK cells provide much promise across a range of contexts. However, there is a fine balance between effective and detrimental NK cell-mediated immune responses and careful consideration is needed when investigating adaptive NK cells and the host response. With the advent of and improved access to genomic technology, we will continue to gain a greater understanding of adaptive NK cells allowing us to further unlock the mysteries of the important role CMV plays in dictating NK cell fate (Figure 2).

## Abbreviations

ALL	Acute lymphoblastic leukemia
AML	Acute myeloid leukemia
ADCC	Antibody-dependent cellular cytotoxicity
CMV	Cytomegalovirus
EBV	Epstein-Barr virus
HIV	Human immunodeficiency virus
HLA	Human leukocyte antigen
HSCT	Hematopoietic stem cell transplantation
HSV	Herpes simplex virus
IFNγ	Interferon gamma
IL	Interleukin
KIR	Killer immunoglobulin-like receptor
LIR	Leukocyte-immunoglobulin-like receptor
MCMV	Murine cytomegalovirus
NK	Natural killer cell
PLZF	Promyelocytic leukemia zinc finger
SOT	Solid organ transplantation
TNFα	Tumor necrosis factor
UCB	Umbilical cord blood
